# Regulatory T Cells in Hepatocellular Carcinoma: Spatial Niches, Biomarkers, and Clinical Implications

**DOI:** 10.3390/ijms27104630

**Published:** 2026-05-21

**Authors:** Dimitris Liapopoulos, Panagiotis Sarantis, Georgios Zogas, Eleni-Myrto Trifylli, Thaleia-Eleftheria Bousou, Konstantina Kamitaki, Ioanna A. Anastasiou, Stefania Kokkali, Sotiris Mavromatis, Evangelos Koustas, Ioannis Elefsiniotis, Theodora Biniari, Michalis V. Karamouzis

**Affiliations:** 1 Biopathological Laboratory, General and Oncology Hospital “Agioi Anargyroi”, National and Kapodistrian University of Athens, Timiou Stavrou 14, 145 64 Kifisia, Greece; dimitrisliapop@gmail.com (D.L.); s.mavromatis@bioanalytica.gr (S.M.); biniaridora@yahoo.com (T.B.); 2 Biopathological Laboratory, Athens Medical Group, Psychiko Clinic, Antersen 1, 115 25 Psychiko, Greece; 3 Department of Biological Chemistry, Medical School, National and Kapodistrian University of Athens, Mikras Asias 75, 115 27 Athens, Greece; 4 University Clinic of Internal Medicine, General and Oncology Hospital “Agioi Anargyroi”, National and Kapodistrian University of Athens, Timiou Stavrou 14, 145 64 Kifisia, Greece; gzogas99@gmail.com (G.Z.); ielefs@nurs.uoa.gr (I.E.); mkaramouz@med.uoa.gr (M.V.K.); 5 Institute of Molecular Medicine and Biomedical Research, 115 27 Athens, Greece; 6 Second Department of Internal Medicine-GI-Liver Unit, General Hospital ‘Hippokratio’, Medical School, National and Kapodistrian University of Athens, Vasilissis Sofias 114, 115 27 Athens, Greece; elenimyrto.trif@gmail.com; 7 First Department of Internal Medicine, Laiko General Hospital, Agiou Thoma 17, 115 27 Athens, Greece; thaliaelb@yahoo.com; 8 Blood Bank, General and Oncology Hospital “Agioi Anargyroi”, National and Kapodistrian University of Athens, Timiou Stavrou 14, 145 64 Kifisia, Greece; k_kamitaki@yahoo.gr; 9 Department of Pharmacology, Medical School, National and Kapodistrian University of Athens, Mikras Asias 75, 115 27 Athens, Greece; anastasiouiwanna@gmail.com; 10 Oncology Unit, Second Department of Medicine, University of Athens, Hippocratio General Hospital of Athens, 115 27 Athens, Greece; stefaniakokkali8@gmail.com; 11 Oncology Department, General Hospital Evangelismos, Ipsilantou 45-47, 106 76 Athens, Greece; vang.koustas@gmail.com

**Keywords:** regulatory T cells, hepatocellular carcinoma, tumor microenvironment, CCR8, ICOS, adenosine pathway, spatial biomarkers, immunotherapy

## Abstract

Hepatocellular carcinoma (HCC) is a leading cause of cancer mortality worldwide, increasingly driven by metabolic dysfunction-associated steatotic liver disease alongside viral and alcohol-related cirrhosis. The tolerogenic immune environment of the liver enables tumor immune escape, with regulatory T cells (Tregs) playing a central role. This review synthesizes human-focused evidence (tissues, blood, clinical cohorts, and single-cell/spatial studies) through September 2025 to define how Tregs are recruited, maintained, and functionally deployed in HCC. Across datasets, intratumoral effector-like Tregs (eTregs) expressing ICOS, CTLA-4, CCR8, and CD39/CD73 accumulate within tumors and co-localize with exhausted cytotoxic PD-1^hi^ CD8^+^ T cells and suppressive myeloid cells. Recruitment is driven mainly by CCL20–CCR6 and CCL22/CCL17–CCR4 signaling, while CCR8 marks highly suppressive tumor-resident Tregs. Their persistence is supported by TGF-β, IL-10, IL-35, adenosine signaling, IL-2 sequestration, and metabolic adaptation. Spatial biomarkers, including ICOS^+^/CCR8^+^ eTreg density and CD8:Treg ratios, associate with prognosis and emerging immunotherapy responses. Etiology further shapes immune architecture: HBV-related HCC often forms Treg-exhausted T-cell niches around viral antigens, whereas MASLD/MASH promotes stromal and metabolic barriers that may reduce PD-(L)1 efficacy. Current treatments (PD-(L)1 blockade with anti-VEGF or CTLA-4, and some TKIs) intersect with Treg biology, while emerging strategies targeting CCR8, CCR4, ICOS, or the adenosine pathway aim to selectively disrupt intratumoral eTreg networks. This review underscores that an etiology-aware, spatial-biomarker framework may guide the integration of selective Treg targeting with PD-(L)1-based therapies in HCC.

## 1. Introduction

Hepatocellular carcinoma (HCC) accounts for the vast majority of primary liver cancers and remains a leading cause of cancer mortality worldwide, reflecting a confluence of viral, toxic, and metabolic drivers layered upon chronic liver injury and cirrhosis [1,2]. While the global burden continues to expand, its geographic heterogeneity mirrors etiologic patterns, HBV predominates in East Asia and sub-Saharan Africa, HCV and alcohol have stronger footprints in parts of Europe and North America, and metabolic dysfunction-associated steatohepatitis (MASH), the progressive form within the newly defined metabolic dysfunction-associated steatotic liver disease (MASLD) spectrum, is rising everywhere [2,3]. Late disease presentation and diagnostic time, as well as limited access to high-quality surveillance, attenuate the efficacy of curative modalities, anchoring the disproportionate contribution of HCC to cancer deaths [1,4] Meanwhile, surveillance gaps are persistent and multifactorial even in well-resourced systems, with many at-risk individuals (particularly those with nonviral cirrhosis) not being recognized or inconsistently surveilled, leading to stage migration at diagnosis and diminished eligibility for resection, ablation, or transplantation [4]. As MASLD/MASH becomes a dominant substrate for chronic liver disease, surveillance will have to adapt to metabolic phenotypes, mixed etiologies (e.g., metabolic plus alcohol-related disease (ALD), the so-called metALD), and the reality that many patients lack specialist contact [3]. 

Constant exposure to gut-derived antigens conditions hepatic sinusoidal endothelial cells, Kupffer cells, stellate cells, and hepatocytes to present antigens in immunosuppressive contexts (e.g., IL-10, PD-L1), promoting deletion or anergy rather than full effector activation [5]. In this environment, regulatory CD4^+^ T cells (Tregs; canonically CD4^+^FOXP3^+^) acquire specialized phenotypes shaped by hepatic stromal signals and metabolic adaptation to hypoxic, nutrient-poor, and steatotic conditions. While this intrinsic tolerance protects against excessive inflammation from continuous gut-derived signals, it also creates a microenvironment that tumors can readily exploit for immune evasion in HCC. The importance of Tregs accumulation in HCC tissue and peripheral blood has to be underscored, as they suppress antitumor immune response (cytotoxic T-cell activity) and are associated with poorer clinical outcomes. 

Three converging developments motivate the present synthesis. First, single-cell and spatial-transcriptomic atlases of human HCC now resolve regulatory T cells (Tregs) at a granularity that was unattainable only a few years ago, revealing that not all FOXP3^+^ cells are equivalent targets and that a distinct, intratumoral effector Treg (eTreg) niche dominates the suppressive landscape. Second, the epidemiological center of gravity of HCC is shifting: MASLD/MASH is overtaking viral hepatitis as the fastest-growing substrate, and emerging data suggest that MASH-related HCC may respond differently to PD-(L)1 blockade than viral HCC, raising the possibility that Treg biology and trial design should be stratified by etiology rather than treated as uniform. Third, the therapeutic pipeline has begun to translate Treg biology into tumor-selective agents—Fc-optimized anti-CCR8 antibodies, anti-CCR4 depleters, ICOS-guided modulators, and adenosine-axis inhibitors are now in first-in-human testing, creating a practical need for biomarkers that can identify which patients harbor the suppressive niches these agents aim to dismantle. In this review, we integrate insights from liver tolerance biology with high-resolution maps of Treg states and niches in HCC, linking these to clinical end points such as prognosis and treatment responsiveness, and underscoring their translational implications. Our overarching aim is to move from description to deployment by aligning spatially resolved Treg biology with rational, testable strategies in human HCC. Throughout, we prioritize human evidence; murine data are cited only where they inform mechanism in the absence of comparable human studies, and are explicitly flagged as such to avoid conflation.

## 2. The Implication of Tregs in the Hepatic Immune System Versus HCC 

### 2.1. The Role of Tregs in the Hepatic Immune System 

Intrinsic immune tolerance of the liver is physiologically adaptive, protecting it against several constant inflammatory signals from the gut microbiome or food, with several non-parenchymal/parenchymal and immune cells, interacting for homeostasis maintenance. However, this microenvironment can also be readily exploited for tumoral immune evasion. Within this tolerogenic landscape, regulatory T cells (Tregs) are pivotal. In humans, Tregs are classically defined as CD4^+^FOXP3^+^ cells. Yet, two caveats are essential for translational work: (i) FOXP3 can be transiently induced in activated conventional T cells (Tconv), so FOXP3 positivity alone does not guarantee suppressive identity; and (ii) Helios, often used as a thymic-Treg surrogate, marks stability more than ontogeny and is not a definitive lineage discriminator [6,7]. A more rigorous criterion for lineage identity in humans is the epigenetic status of the Treg-specific demethylated region (TSDR), an evolutionarily conserved CpG-rich element within the FOXP3 locus (CNS2 / intron 1) whose complete demethylation is restricted to stable, bona fide Tregs and is not reproduced by TGF-β-induced or activation-induced FOXP3^+^ Tconv cells [8,9]. Demethylation at the TSDR is tightly coupled to FOXP3 expression stability under inflammatory pressure and correlates with suppressive function more reliably than FOXP3 protein level alone; it is therefore increasingly regarded as the gold standard for defining “lineage” Tregs in translational studies [8,9]. Importantly for HCC, the vast majority of published tumor single-cell and flow-cytometry datasets rely on FOXP3 protein (with or without CD25 co-staining) and very few incorporate TSDR methylation readouts on sorted intratumoral FOXP3^+^ cells. This is a non-trivial limitation: some “Tregs” scored in HCC cohorts—especially at inflamed peritumoral or portal-tract interfaces—may represent transiently FOXP3^+^ activated Tconv rather than stable suppressive cells, which could partly explain the heterogeneous associations between bulk FOXP3^+^ counts and outcome. Integrating TSDR methylation (or its flow-cytometric surrogates, such as CD45RA/FOXP3 co-staining combined with CTLA-4, CD39 and CCR8) with composite phenotypes is therefore preferable whenever tissue permits, and should become a standard in future HCC biomarker studies that aim to guide Treg-directed therapy.

Additionally, there are two functionally distinct Treg states, which are relevant in cancer, including human CD45RA^+^FOXP3^lo^ “resting” (rTreg) and CD45RA^−^FOXP3^hi^ “effector” (eTreg) fractions, which differ in proliferative history, cytokine competence, and suppressive potency [10]. 

Moreover, tumors preferentially expand/retain eTregs characterized by high IL-2 receptor expression and activation/inhibitory modules (e.g., CTLA-4, ICOS) and ectonucleotidases (CD39), features consistently observed in HCC single-cell maps [10,11]. Single-cell atlases of human HCC consistently recover intratumoral Treg clusters enriched for activation and suppressive modules, providing an entry point to dissect their phenotype, induction, and fitness within a tolerogenic organ [12,13], while these adaptations converge to produce an effector-like Treg (eTreg) state that is highly suppressive and clinically relevant. Single-cell and spatial studies consistently show Treg-rich intratumoral niches and unfavorable CTL:Treg ratios, with ICOS^+^/CTLA-4^+^/CD39^+^ eTregs positioned to inhibit antitumor immunity. Lastly, Tregs also contribute to immunometabolic immune escape (e.g., adenosine signaling) and intersect with current therapies, although clinically meaningful Treg modulation in patients remains unclear.

### 2.2. The Role of Tregs in HCC Tissue

There are four main reasons why Tregs are important in HCC. First, they accumulate in the tumors and blood of patients with HCC and are associated with impaired cytotoxic T lymphocyte (CTL) activity and poorer clinical outcomes, as shown in early human studies [14]. Second, single-cell and spatial analyses across different etiologies (HBV, HCV, MASLD/MASH) consistently reveal Treg-enriched intratumoral niches and unfavorable CTL:Treg ratios, with ICOS^+^/CTLA-4^+^/CD39^+^ eTreg clusters positioned to suppress cytotoxic responses [11,13]. Third, Tregs are linked to immunometabolic pathways that reinforce immune escape, particularly through adenosine production via CD39/CD73 signaling, which suppresses effector T cells and has been associated with resistance to anti-PD-1 therapy [15]. Fourth, current therapies for advanced HCC also intersect with Treg biology: anti-PD-L1 plus anti-VEGF (atezolizumab–bevacizumab) improves survival compared with sorafenib and alters angiogenic–immune interactions, while CTLA-4-based regimens (tremelimumab plus durvalumab) target pathways central to Treg function [16,17].

The extent to which these treatments achieve meaningful intratumoral Treg modulation in patients remains unclear and will require evaluation using spatially resolved biomarkers. Notably, intratumoral enrichment of ICOS^+^FOXP3^+^ Tregs has been associated with reduced survival in patients with HCC, further highlighting the clinical relevance of eTreg skewing [18]. Treg biology in the liver reflects an inherent tolerogenic default, operationalized through LSEC, Kupffer-cell, and HSC programs that encourage Treg induction and stability, and an effector-like Treg (eTreg) phenotype that embraces adenosine signaling, costimulatory rewiring, and metabolic adaptations keyed to lactate and lipid handling [18]. 

#### 2.2.1. Phenotypic Profile of Tumor-Infiltrating Tregs in HCC

Phenotypically, human tumor-resident Tregs in multiple cancers, including HCC, share a core eTreg signature marked by high CTLA-4, ICOS, and the ectoenzymes CD39 (ENTPD1) and CD73 (NT5E), with chemokine receptor skewing that favors CCR8 and CCR4 expression [12,19,20]. CCR8 in particular has emerged as a hallmark of human intratumoral eTregs with potent suppressor function, providing an attractive therapeutic target [20]. Within HCC, scRNA-seq datasets identify ICOS^+^/CTLA-4^+^ Treg clusters with elevated CCR8, cementing the concept of a distinct eTreg niche that co-localizes with exhausted cytotoxic T cells and immunosuppressive myeloid populations [12,13]. The adenosinergic axis is integral to this phenotype: CD39/CD73 on Tregs hydrolyze extracellular ATP to adenosine, engaging A2A receptors on effector cells and dampening antitumor immunity [21].

#### 2.2.2. eTreg State Maintenance and Hepatic Stromal Induction

The hepatic stromal microenvironment imprints and maintains this eTreg state via three non-parenchymal cell types that have a central role, including (i) liver sinusoidal endothelial cells (LSECs), Kupffer cells, and hepatic stellate cells (HSCs). Human LSECs present an antigen under low co-stimulation and abundant inhibitory ligands. Experimental models demonstrate that LSECs drive de novo Foxp3 induction and stabilize Treg identity via membrane-bound TGF-β signaling, recapitulating a tolerogenic licensing program that is well suited to the hepatic environment [22]. 

Meanwhile, Kupffer cells, the resident macrophages of the liver, amplify this program by releasing IL-10 and presenting TGF-β in response to apoptotic cargo, thereby biasing local CD4^+^ T-cell differentiation toward Foxp3^+^ fates and blunting effector responses [23]. On top of that, HSCs, vitamin A-rich perisinusoidal cells that remodel extracellular matrix, add a third layer by producing retinoic acid (RA), which synergizes with TGF-β to promote Foxp3 induction and Treg stability; human and murine data show HSC-derived RA preferentially expands Foxp3^+^ cells over conventional T cells [24]. 

Together, these stromal circuits deliver a combination of TGF-β, IL-10, and RA that consolidates a Treg-permissive differentiation trajectory at baseline and is co-opted within HCC nodules. Nevertheless, cytokine milieus further consolidate Treg function in the liver. In addition to TGF-β and IL-10, interleukin-35 (IL-35), a heterodimeric cytokine, is secreted by Tregs and other cells and has been linked to human HCC biology. IL-35 expression is closely associated with aggressiveness and an earlier post-operative recurrence, while it is also correlated with the infiltration by CD39^+^FOXP3^+^ Tregs, indicating a feed-forward loop between IL-35 and adenosine-competent eTregs [25]. These axes (LSEC-TGF-β, Kupffer-IL-10/TGF-β, HSC-RA, and Treg-derived IL-35) are not redundant; rather, they are layered to ensure both induction and phenotypic stability of Tregs in the face of inflammatory flux.

#### 2.2.3. Metabolic Adaptations of Hepatic Tregs

Tregs must function in environments defined by intermittent hypoxia, high lactate, and lipid overload, features exacerbated in steatohepatitis and HCC, implying the necessity of metabolic adaptations, accordingly. 

FOXP3 itself rewires T-cell metabolism by suppressing MYC-driven glycolysis and promoting oxidative phosphorylation (OXPHOS), granting Tregs a fitness advantage in low-glucose, high-lactate niches typical of tumors and ischemic cirrhotic tissue [26]. In human tumors, lactic acid uptake through MCT1 drives NFAT1-dependent PD-1 expression specifically in eTregs, reinforcing their suppressive program and even contributing to resistance to PD-1 blockade in glycolytic cancers, including liver tumors [27]. 

Complementing this lactate tolerance, Tregs preferentially engage fatty-acid oxidation (FAO) to sustain function and survival; experimental systems demonstrate that FAO complements glycolysis in Tregs and that perturbing this balance compromises suppressive capacity [28]. A mechanistic lynchpin is CD36: intratumoral Tregs upregulate this fatty-acid transporter to import long-chain lipids, activate PPAR-β signaling, and bolster mitochondrial fitness in the acidic TME; genetic or pharmacologic interference with CD36 selectively diminishes tumor Tregs and augments antitumor immunity without global autoimmunity in preclinical models [29]. Together, these data argue that hepatic eTregs are metabolically specialized cells optimized for an HCC-like microenvironment that is acidic, hypoxic, and lipid-rich.

Beyond lactate, the steatotic liver generates a broader portfolio of metabolic byproducts that plausibly shape Treg signaling, although direct mechanistic links in human HCC remain under-characterized. Oxidized and saturated free fatty acids (notably palmitate) accumulate in MASLD/MASH livers and alter T-cell bioenergetics; in parallel, Tregs upregulate CD36 and PPAR-β programs that allow them to tolerate lipotoxic stress better than effector cells, translating lipid overload into a relative fitness advantage [29]. Bile acids also warrant mention: secondary bile acids such as isoallo-lithocholic acid and 3-oxoLCA have been shown in non-hepatic systems to modulate FOXP3 induction and Th17/Treg balance via nuclear receptors (FXR, TGR5, RORC) [30], and MASH-associated dysbiosis reshapes the bile-acid pool in ways that could bias hepatic T-cell differentiation toward Treg-permissive states. Short-chain fatty acids (SCFAs) of microbial origin, delivered through portal circulation, promote peripheral Treg induction via HDAC inhibition and GPR43 signaling in experimental systems [31]; whether this axis is amplified, blunted, or qualitatively rewired in MASH-related HCC is not yet resolved. Reactive oxygen species generated during hepatocellular lipotoxicity, kynurenine-pathway metabolites downstream of IDO1 (expressed by tolerogenic myeloid and stromal cells), and succinate accumulating in hypoxic cores represent further candidate inputs that could sustain Treg suppressive programs while impairing effector function. We emphasize that most of these links have been established in non-hepatic tumors or in murine MASH models; their relative magnitude and tissue compartmentalization in human HCC remains an open question that should be prioritized in future spatially resolved metabolomic studies.

#### 2.2.4. Treg Markers in HCC Tissues

It is worth pausing on the specificity of human Treg markers when translating from mouse to HCC. Neuropilin-1 (NRP1), frequently used to distinguish thymic (tTreg) from peripherally induced (pTreg) cells in mice, is not a specific marker of human Tregs, behaving more like an activation antigen across T-cell subsets; thus, it should not be used to define human HCC Tregs [32]. Helios, likewise, is better considered a correlate of activation/stability rather than a lineage determinant in human Tregs. Moreover, CRISPR ablation studies show that Helios is a marker, not a driver, of human Treg stability, and it can be induced outside the thymus [7,33]. By contrast, convergent human tumor datasets support eTreg hallmarks such as CCR8, ICOS, CTLA-4, and the CD39/CD73 adenosine machinery, features repeatedly recovered in human HCC single-cell maps and pan-cancer Treg compendia [12,19,20]. Lastly, human HCC harbors chemokine and metabolic niches that favor CCR8^+^ICOS^+^CD39^+^ eTregs and harnesses stromal and cytokine cues to maintain them. The practical implication is clear: therapeutic strategies that respect the human-specific marker caveats (eschewing NRP1/Helios as defining antigens) and instead target validated eTreg features (CCR8/CCR4, CTLA-4/ICOS modules, CD39/CD73, PD-1 induction by lactate, and CD36-dependent FAO) are most likely to translate in HCC [19,20,21,27,29]. The next section will therefore focus on how these cells arrive and persist in the tumor, chemokines, maintenance circuits, and the suppressive mechanisms they deploy in human HCC.

## 3. Mechanisms of Recruitment and Maintenance

If HCC is a highly immunosuppressive tumor in a tolerogenic organ, Tregs must be actively recruited and then sustained within it. Human data pinpoint three chemokine axes that most convincingly explain how Tregs get there: CCL20-CCR6, CCL22/CCL17-CCR4, and CCR8 (largely via CCL1). Single-cell maps from resected human HCC already hinted at an expanded, activated intratumoral Treg state adorned with chemokine receptors and ectonucleotidases, providing a mechanistic bridge to the maintenance pathways that keep them on site and suppressive [12].

### 3.1. Chemokine-Mediated Recruitment

The first such axis is CCL20-CCR6. Tumor and peritumoral cells in HCC can produce CCL20, while a proportion of human Tregs display CCR6, creating a gradient that preferentially recruits them into the lesion. In human cohorts, higher CCL20 expression and CCR6^+^ Tregs associate with more aggressive disease, anchoring this axis in clinical material [34]. Therapeutic rationale: Neutralizing CCL20 or functionally blocking CCR6 may reduce the initial Treg influx; although not yet tested in HCC trials, this axis has an intuitive combinatorial logic with PD-(L)1 backbones given its upstream recruitment positioning [34].

A second axis is CCL22/CCL17-CCR4. Among the strongest human HCC datasets, single-cell and spatial profiling reveal ICOS^+^, CCR4^+^ effector Tregs concentrated in intratumoral niches that also harbor chemokine-expressing myeloid cells (notably macrophages and dendritic cells), consistent with a CCL22/CCL17-driven recruitment loop [35]. Independent clinical pathology work further shows that CCR4 expression in HCC is linked to invasive behavior and metastasis, underscoring the biological and prognostic salience of this receptor in the liver setting [36]. Therapeutic rationale: CCR4-directed antibodies (and next-generation depleters) could selectively thin intratumoral eTregs, an approach conceptually attractive in cirrhotic patients if intratumoral bias can be preserved to mitigate systemic autoimmunity [35,36].

A third axis, engaged largely via CCL1, operates through CCR8. Multiple tumor types enrich for CCR8 on effector Tregs; human HCC scRNA-seq/spatial analyses report a CCR8-high Treg program within tumors, aligning with the broader literature that places CCR8 on highly suppressive, activated eTregs [12]. In liver cancer models, pharmacologic CCR8 antagonism reduces CCR8^+^ Tregs, remodels the microenvironment, and augments antitumor immunity, nominating CCR8 as a tractable handle for intratumoral Treg depletion in HCC [37]. Therapeutic rationale: CCR8 antibodies/antagonists, alone or combined with PD-1/VEGF backbones, represent a rational next wave for HCC built on a tumor-selective Treg marker [12,37] (Table 1). 

### 3.2. Intratumoral Maintenance Signals

Once recruited, maintenance pathways entrench Tregs and sustain their suppressive tone.

TGF-β provides canonical inductive cues. Human HCC tissue shows that tumor-derived TGF-β1 promotes Treg accumulation; mechanistically, HCC cells can induce Treg differentiation and stability via TGF-β signaling, with higher TGF-β correlating to poor outcomes [38]. This provides a canonical inductive axis that not only generates Tregs but also stabilizes FOXP3 expression and suppressive programs within hypoxic, fibrotic tumor beds [38].

IL-10 reinforces this maintenance program. Hepatoma-cell-derived IL-10 enhances human Treg stability via JAK1/STAT5, countering inflammatory erosion of FOXP3 and boosting Treg persistence/function in vitro, mechanistically aligning with maintenance rather than pure recruitment [39]. Given the abundance of IL-10-rich myeloid niches in HCC, this pathway likely reinforces Treg programs after entry [39]. 

IL-35 contributes a further suppressive layer. Human HCC studies report elevated IL-35 with Treg-linked immunosuppression, positioning IL-35 as both a product and a sustainer of suppressive circuits within tumors [40]. 

Adenosine signaling, generated by CD39 and CD73 and acting through A2A receptors, completes the metabolic arm of maintenance. HCC Tregs frequently express CD39, and higher CD39^+^ Treg frequency associates with adverse prognosis, consistent with adenosine accumulation that dampens effector T-cell function via A2A signaling [41]. In orthotopic liver cancer models with ancillary human data, A2A receptor blockade synergizes with anti-PD-1 to shrink tumors, providing a compelling mechanism to disrupt maintenance to combine with checkpoint therapy [42]. Therapeutic rationale: Adenosine-axis inhibitors (A2A antagonists; CD73/CD39 blockers) layered onto PD-(L)1±anti-VEGF are well justified in HCC-like TMEs [41,42]. 

IL-2 sequestration by high-CD25 Tregs operates as a fifth axis. High CD25 expression enables Tregs to scavenge local IL-2, starving effector T cells and sustaining Treg survival; while demonstrated broadly in cancer immunology, this mode is highly plausible in the cytokine-poor, hypoxic niches of HCC [43]. Therapeutic rationale: Strategies that “shield” IL-2 from Tregs (e.g., IL-2 muteins biased to effector cells) merit testing alongside HCC backbones [43] (Table 1). 

### 3.3. Mechanisms of Antitumor Immune Response Suppression

These maintenance inputs dovetail with mechanisms of suppression observed in HCC.

CTLA-4-linked suppression is the most prominent of these. HCC patients harbor increased GARP^+^CTLA-4^+^FOXP3^+^ Tregs. This phenotype marks highly suppressive, activated Tregs and correlates with impaired T-cell functionality [44]. Beyond checkpoint ligation, CTLA-4 can remove CD80/CD86 from APCs via trans-endocytosis, throttling co-stimulation. Therapeutic rationale: Intratumoral Treg depletion via CTLA-4-engaging agents likely contributes meaningfully to clinical benefit in HCC regimens that include CTLA-4, but optimizing selectivity remains critical in cirrhosis [44].

Cytokine-mediated suppression operates in parallel. TGF-β, IL-10, and IL-35 produced by (or acting on) Tregs suppress cytotoxic programs and antigen presentation; in HCC, tumor- and myeloid-derived TGF-β/IL-10 reinforce Treg circuits while directly dampening Teff cells [38,39,40]. Therapeutic rationale: TGF-β traps or bifunctional PD-L1/TGF-β blockers could be paired with PD-(L)1 to erode cytokine maintenance and restore antitumor immunity [38].

Metabolic competition and adenosine signaling close the loop. CD39/CD73-driven adenosine flux and IL-2 consumption fit the hypoxic, lactate-rich ecology of HCC, curtailing Teff proliferation and function while stabilizing Tregs [41,43]. Therapeutic rationale: A2A antagonists and CD73/CD39 inhibitors can be layered on PD-(L)1±anti-VEGF to rewire nutrient and purinergic landscapes [41,42].

**Figure 1:**
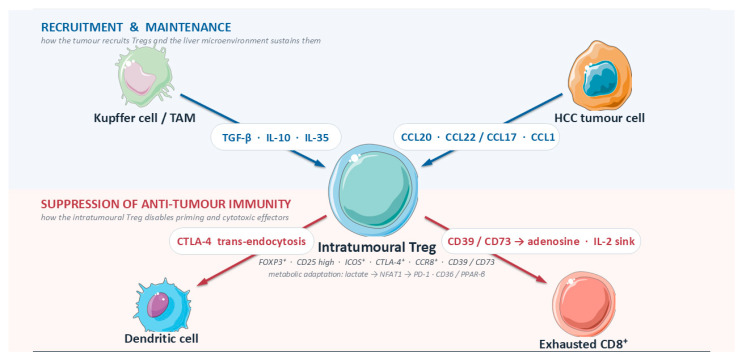
Regulatory T cells in the HCC microenvironment—mechanisms of immune escape. Schematic representation of how hepatocellular carcinoma (HCC) recruits and sustains intratumoral regulatory T cells (Tregs) and how these Tregs suppress anti-tumor immunity. Kupffer cells and tumor-associated macrophages (TAMs) secrete TGF-β, IL-10, and IL-35, while HCC tumor cells release CCL20, CCL22/CCL17, and CCL1 to promote Treg recruitment and maintenance. Intratumoral Tregs (FOXP3^+^, CD25^high, ICOS^+^, CTLA-4^+^, CCR8^+^, CD39/CD73^+^) inhibit dendritic-cell priming via CTLA-4-mediated trans-endocytosis and suppress cytotoxic CD8^+^ T-cell activity through adenosine production (CD39/CD73 pathway) and IL-2 consumption. Metabolic adaptation (lactate → NFAT1 → PD-1, CD36, PPAR-δ) further enhances Treg persistence. Clinically, exhausted CD8^+^ T cells (PD-1^high TIM-3^+^ LAG-3^+^) and low intratumoral CD8/Treg ratios correlate with shorter survival, earlier recurrence, and reduced response to immune-checkpoint blockade. Cell illustrations adapted from Servier Medical Art (CC BY 3.0).

Collectively, chemokine-guided recruitment puts Tregs in the right place (CCR6/CCR4/CCR8), maintenance signals keep them alive and dominant (TGF-β, IL-10, IL-35, adenosine, IL-2 capture), and suppression mechanisms neutralize antitumor circuits (CTLA-4 programs, cytokines, purinergic brakes). The therapeutic levers are correspondingly modular: block entry (CCR4/CCR8, CCR6), remove metabolic support and inhibitory signals (A2A/CD39/CD73; IL-2 bias), and selectively deplete intratumoral eTregs (CCR8- or CCR4-directed depletion; CTLA-4-containing regimens). Human HCC data already justify each module; the translational task now is rational combination and spatially aware patient selection [12,35,37,41,42] (Figure 1). 

## 4. Spatial Distribution and Clinical Significance

Single-cell atlases of human HCC provided the first high-resolution view that Tregs are not simply “present” but transcriptionally specialized and clonally enriched within tumor beds. In the seminal HCC T-cell scRNA-seq, intratumoral Tregs displayed a distinct activation/exhaustion program (e.g., LAYN, CTLA-4, TIGIT) and clonal expansion relative to peritumor or blood, arguing for bona fide tumor adaptation rather than passive influx [12]. An expanded pan-immune single-cell map later confirmed stable Treg states across HCC patients, with coordinated shifts in other lymphoid and myeloid compartments, implying that Treg accumulation is embedded in broader TME programs [11]. Spatially, multiplex/spatial assays reveal that ICOS^+^ eTregs preferentially occupy intratumoral niches (including tumor center) rather than peritumor, aligning with a suppressive geography that coincides with cytotoxic T-cell scarcity [18,45]. 

### 4.1. Intratumoral Localization

These spatial patterns carry prognostic meaning when converted into ratios. Classic pathology studies established that the intratumoral balance between cytotoxic CD8^+^ T cells and Tregs, more than either density alone, predicts recurrence and overall survival after resection [46]. Contemporary single-cell/spatial datasets now rationalize that observation: tumors with enriched eTreg clusters show depressed effector programs and TCR clonotypes consistent with exhaustion, while peritumor retains comparatively “healthier” cytotoxic states [11,12]. In practical clinicopathologic terms, low intratumoral CD8/Treg or PD-1^+^CD8:ICOS^+^Treg ratios portend inferior outcomes; conversely, higher effector-skewed ratios associate with better survival and, in some cohorts, better immunotherapy responsiveness [46,47].

### 4.2. Prognostic Value of Treg Subsets Localization

Which Tregs matter most spatially? Both scRNA-seq and IHC/spatial immunophenotyping repeatedly highlight ICOS^+^, CTLA-4^+^, and CCR8^+^ eTregs as the intratumoral subset with strongest suppressive signatures. ICOS^+^ FOXP3^+^ Tregs are overrepresented in HCC tumors and independently associate with worse survival, dovetailing with spatial observations that these cells concentrate within tumor regions [18,45]. CCR8, a canonical ti-Treg marker across cancers, is increased on human HCC TIL-Tregs and marks the most suppressive states; in preclinical HCC, CCR8 antagonism remodels Tregs toward a less immunosuppressive phenotype and expands intratumoral CD8^+^ cytotoxicity, supporting CCR8^+^ eTregs as spatially and functionally central [37]. These CCR8^+^/ICOS^+^ hubs likely represent “immunosuppressive nodes” that are physically proximate to dysfunctional CD8^+^ T cells, myeloid PD-L1^+^ cells, and vasculature, jointly shaping immune privilege within tumor nests [45,48]. 

On the CD8 side of the ratio, HCC is enriched for PD-1^hi^ exhausted CD8^+^ T cells that cluster near immunosuppressive elements in situ. Multi-cohort analyses show PD-1^hi^ (especially TIM-3^+^PD-1^hi^) CD8^+^ T cells have inferior effector function and worse prognosis, and spatial analyses place them in proximity to PD-L1^+^ tumor-associated macrophages, precisely where ICOS^+^ eTregs are abundant [45,48]. This convergence motivates compound spatial metrics: for example, a high PD-1^+^CD8:high ICOS^+^Treg ratio has been linked to improved response to immune checkpoint blockade in HBV-related HCC, whereas a low ratio tracks with refractoriness [47]. As such, spatially anchored “effector-to-regulator” indices may outperform bulk counts and deserve development as companion diagnostics.

### 4.3. Etiology-Dependent Immune Topography

Etiology modifies these spatial/functional relationships. In HBV-related HCC, multimodal single-cell/epigenomic work demonstrates that HBV-specific T cells and HBV-related Treg subsets share core regulatory programs (NF-κB/NFAT/NR4A axes), with HBV-related Treg-CTLA4 populations clonally enriched in tumors; HBV viral load correlates with tumor-infiltrating Tregs and poorer outcomes [49]. Mechanistically, the co-selection of suppressive Treg and exhausted CD8 programs around HBV antigens can be parsed into three interlocking transcriptional circuits. Chronic TCR engagement by persistent viral peptides drives sustained calcium flux and nuclear translocation of NFAT; in the absence of balanced AP-1 partnering, NFAT acts in a “partnerless” configuration that activates an exhaustion-and-tolerance gene program, including PD-1, CTLA-4, TIGIT and LAG-3 in CD8 cells and reinforcement of FOXP3 and CTLA-4 in Tregs [50]. The same chronic signaling induces the NR4A family (NR4A1/Nur77, NR4A2/Nurr1, NR4A3/NOR1), orphan nuclear receptors that co-operate with NFAT and TOX to lock in the exhausted/regulatory chromatin state and are now recognized as master regulators of T-cell dysfunction in chronic viral infection and cancer [51,52]. In parallel, non-canonical NF-κB signaling downstream of HBV-driven inflammatory cues (TNFRSF members, LTβR) and canonical NF-κB activity triggered by HBx-modulated innate sensing contribute a Treg-stabilizing input: c-Rel binding at the CNS2/TSDR and proximal FOXP3 enhancer reinforces lineage commitment and suppressive function [52]. Because these three axes (NFAT, NR4A, NF-κB/c-Rel) converge on the same FOXP3-enhancer architecture and on shared CTLA-4/PD-1 loci, HBV-specific T-cell clones and HBV-context Tregs end up sharing large portions of their regulatory landscape—an observation directly supported by the epigenomic data of You et al. [49]. This shared wiring helps explain why intratumoral niches in HBV-HCC so consistently co-localize CTLA-4^+^ Tregs with PD-1-high exhausted cytotoxic CD8^+^ T cells around viral-antigen-rich regions, and it suggests that agents which target the NR4A/TOX axis or disengage partnerless NFAT could, in principle, reprogram both populations simultaneously, a hypothesis that deserves formal testing in HBV-HCC-specific model systems. These findings imply that viral antigenic context co-selects Treg and exhausted CD8 states within intratumoral niches, potentially explaining why some HBV-HCCs display particularly suppressive Treg neighborhoods yet retain antigen-linked opportunities for reversal [11,49]. By contrast, non-viral (especially MASH-related) HCC shows different immune topology: despite abundant PD-1^+^ CD8^+^ cells, antitumor surveillance is impaired, and clinical benefit from anti-PD-(L)1 appears blunted in non-viral aetiologies likely reflecting distinct, tissue-damage-imprinted exhaustion and stromal architectures that Treg hubs can exploit [53]. In other words, HBV may co-organize Treg–Tex niches around viral antigens, whereas MASH organizes immunopathology in ways less permissive to checkpoint reinvigoration even when suppressive cells are present [49,53] (Table 2). 

Clinically, these maps argue for evaluating HCC based not only on “how many Tregs” but “where and which Tregs” relative to effectors and myeloid barriers. Intratumoral enrichment of ICOS^+^/CCR8^+^ eTregs, reduced CD8/Treg or PD-1^+^CD8:ICOS^+^Treg ratios, and spatial proximity of exhausted CD8^+^ T cells to suppressive hubs each forecast poor prognosis and weaker ICI benefit, while the converse predicts more favorable courses [18,46,47]. Importantly, these features are actionable: CCR8-directed strategies aim to selectively dismantle intratumoral eTreg nodes; ICOS-guided depletion concepts target the same clusters; and combinatorial designs can be stratified by spatial ratios to enrich for likely responders [37,45]. As we will discuss in the therapeutic chapter, integrating spatial Treg metrics with etiology-aware enrolment could tighten the link between mechanism and outcome in HCC trials [47,53]. In summary, scRNA-seq and spatial profiling converge on a coherent picture: HCC harbors intratumoral eTreg niches, enriched for ICOS^+^/CCR8^+^/CTLA-4^+^ states, that co-localize with exhausted PD-1^hi^ CD8^+^ T cells and immunosuppressive myeloid elements. These niches compress effector:regulator ratios, predict prognosis, and modulate ICI responsiveness in an etiology-dependent manner. Translationally, “where Tregs are” and “which Tregs they are” should be formalized into spatial biomarkers (e.g., ICOS^+^/CCR8^+^ eTreg density and CD8/Treg or PD-1^+^CD8:ICOS^+^Treg ratios) to guide patient selection and to monitor on-target effects of Treg-directed therapies in HCC [11,12,47].

## 5. Therapeutic Landscape and Translational Pipelines

The treatment paradigm for advanced hepatocellular carcinoma (HCC) has rapidly reorganized around combinations that both release exhausted cytotoxic T cells and dismantle the suppressive niche maintained by regulatory T cells (Tregs). Critically, the current first-line “backbones” already modulate Treg biology, either directly (via CTLA-4) or indirectly (via anti-angiogenic cues that rewire myeloid–stromal states and Treg homeostasis), and a proliferation of translational initiatives now aims to make Treg control more specific (CCR8/CCR4, ICOS-guided depletion) or to collapse their metabolic advantage (adenosine axis). Below, we chart this landscape, anchoring it in HCC trials and human single-cell/spatial data that highlighted ICOS^+^/CCR8^+^ eTreg niches and their clinical correlates [11,13] (Table 3). 

## 6. Impact of Standard-of-Care Regimens

The most widely used regimen, atezolizumab plus bevacizumab, outperformed sorafenib on overall and progression-free survival in IMbrave150 (median OS 19.2 vs 13.4 months in the primary analysis), establishing anti-PD-L1 + anti-VEGF as a standard [16]. Although the trial did not enumerate Tregs, VEGF blockade has long been linked to normalization of vasculature, improved dendritic-cell priming, and constraints on Treg trafficking/maintenance in tumors, all of which plausibly enhance PD-(L)1 reinvigoration in a tolerogenic liver milieu. The second broadly adopted backbone, STRIDE (single priming dose tremelimumab + durvalumab), met non-inferiority and showed OS benefit over sorafenib in HIMALAYA, offering an effective option that uses CTLA-4-mediated Treg modulation alongside PD-L1 blockade [17]. Mechanistically, anti-CTLA-4 can deplete intratumoral Tregs in an FcγR-dependent manner and/or modulate priming in lymphoid organs, effects repeatedly observed across models and patient samples (though antibody isotype and tumor FcγR context matter) [54,55,56]. Importantly for HCC, the STRIDE priming pulse may mitigate continuous CTLA-4 exposure while still perturbing the eTreg niche established in tumors enriched for ICOS^+^/CCR8^+^ Tregs [11]. Even as immunotherapy reshapes HCC care, tyrosine-kinase inhibitors (TKIs) still matter as immune modulators. Sorafenib (SHARP) established the first OS signal for systemic therapy regorafenib (RESORCE), cabozantinib (CELESTIAL), and lenvatinib (REFLECT; non-inferior to sorafenib) expanded options [57,58,59]. Beyond direct antitumor/anti-angiogenic effects, TKIs reshape the TME: preclinical and translational work shows lenvatinib reduces intratumoral Tregs and suppresses TGF-β-associated programs, creating a more ICI-permissive niche and rationalizing synergy with PD-1 [60]. The implication for Treg-centric design is to enrich for tumors where anti-angiogenic pressure specifically intersects eTreg maintenance (e.g., VEGF-high, TGF-β-active, ICOS^+^/CCR8^+^ Treg-rich signatures), as suggested by the lenvatinib+PD-1 gene-signature work [60].

Building on that Treg-aware view of the TME, dual-checkpoint layering leverages CTLA-4’s unique angle. In second line and beyond, nivolumab plus ipilimumab (CheckMate-040 cohort) delivered ~30% objective responses with durable benefit, leading to accelerated approval and widespread off-label adoption in select settings [61]. While not HCC-specific mechanistic readouts, the most credible explanation for PD-1+CTLA-4 synergy includes (i) expansion of novel T-cell clones via CTLA-4-modulated priming; (ii) intratumoral Treg attenuation via Fc-engineered CTLA-4 antibodies where supported by FcγR biology; and (iii) relief of metabolic and cytokine-based suppression that disproportionately affects PD-1^+^CD8^+^ cells in the liver TME [54,55,56]. Notably, Treg-rich spatial niches in HCC (often ICOS^+^/CCR8^+^) have been tied to inferior effector:regulatory ratios and worse outcomes, reinforcing a mechanistic through-line from sc/spatial maps to combination logic [11,13].

## 7. Emerging Treg-Targeted Therapies

With that rationale in hand, Treg-directed strategies are now moving into the clinic. The most mature concept is chemokine-based depletion. CCR4 targeting with mogamulizumab depletes Tregs and has proven activity in CCR4^+^ T-cell malignancies, validating the biology and clinical manageability of systemic Treg depletion (albeit in hematologic disease) [62]. In solid tumors, CCR8 has emerged as a more selective eTreg marker that is highly enriched on intratumoral Tregs, including in HCC single-cell [11]. 

Multiple Fc-optimized anti-CCR8 antibodies show potent, selective Treg depletion and tumor control in preclinical models and are now in first-in-human testing; BAY3375968 exemplifies this class [63,64,65]. GS-1811, similarly engineered for tumor-Treg specificity, underscores how differential CCR8 expression between tumor and normal tissue can enable selective depletion [66]. For HCC, where CCR8^+^ eTregs co-localize with exhausted PD-1^+^CD8^+^ cells and myeloid suppressors, this axis is particularly compelling as an add-on to PD-(L)1 backbones [11].

In parallel, ICOS-guided approaches marry depletion with co-stimulation. ICOS is upregulated on human HCC Tregs and associates with poor prognosis, making it an attractive handle for intratumoral Treg pruning [18]. KY1044, a human IgG1 anti-ICOS, is designed to deplete ICOS^hi^ Tregs while co-stimulating ICOS^lo^ effectors; first-in-human experience and conference reports support the dual-mechanism concept in solid tumors (often in combination with PD-(L)1) [67]. Parallel co-stimulatory agonists (OX40, 4-1BB) may synergize with PD-(L)1 in HCC, but safety is nuanced in cirrhosis: dual OX40+4-1BB agonism (ivuxolimab+utomilumab) was tolerable with preliminary activity in early trials [68], yet historical experience with potent 4-1BB agonists revealed liver-specific toxicities that modern engineering seeks to avoid [69]. If deployed in HCC, such agents should be paired with spatial biomarkers that flag high ICOS^+^/CCR8^+^ Treg niches and monitored for hepatic immune-related adverse events. Rounding out the suppressive circuitry, the adenosine axis offers another lever relevant to Tregs in HCC. Tregs in HCC frequently express the ectonucleotidases CD39 and CD73, feeding into A2A receptor-mediated suppression; intratumoral CD39 also marks dysfunctional but tumor-reactive T cells, highlighting the currency of adenosine in liver tumors [11,70]. From a trial-readiness standpoint, the actionable landscape maps neatly onto these levers. First line: atezo+bev (IMbrave150) and STRIDE (HIMALAYA) are validated backbones with room for Treg-focused add-ons [16,17]. TKI anchors: sorafenib (SHARP), lenvatinib (REFLECT), regorafenib (RESORCE), and cabozantinib (CELESTIAL) remain essential, with translational evidence that lenvatinib+PD-1 reduces Tregs and TGF-β signaling [57,58,59,60,71]. Second line and beyond: PD-1 monotherapy signals (KEYNOTE-224; KEYNOTE-240 failed to cross prespecified thresholds despite numerical benefit) frame combination needs [16,72]. Dual ICI (nivolumab+ipilimumab) offers a clinically meaningful option with a plausible Treg mechanism [61]. In the translational pipeline: CCR8-directed depletion (BAY3375968; GS-1811) and ICOS-guided Treg pruning (KY1044) exemplify tumor-Treg-selective approaches likely to combine best with PD-(L)1 and, in HCC, with anti-VEGF to reinforce vascular and myeloid normalization [65,66,67]. Given the etiologic heterogeneity of HCC and fragile hepatic reserve, programs that (i) preferentially deplete intratumoral (not systemic) Tregs (e.g., CCR8), (ii) avoid broad co-stimulation in inflamed cirrhotic livers, and (iii) use spatial single-cell biomarkers (PD-1^+^CD8: ICOS^+^/CCR8^+^ Treg) to gate entry are poised to translate fastest [11,13].

In short, today’s HCC regimens modulate Treg biology, and the near-term pipeline is converging on precision Treg control. The pragmatic strategy is to keep the proven pillars (PD-(L)1±CTLA-4; VEGF or TKI) and layer in selective Treg-targeting (CCR8/CCR4, ICOS-guided) or adenosine-axis agents for tumors with a documented eTreg/adenosine-rich spatial phenotype, while respecting the safety calculus of cirrhosis [16,17,61,63,65].

## 8. Controversies, Pitfalls and Future Directions

Against this backdrop, markers and measurement turn out to be as important as mechanisms. In human tissues, including HCC, FOXP3 is neither fully specific nor uniformly stable, making “Treg” quantification challenging if based on FOXP3 alone. Activated conventional T cells transiently upregulate FOXP3 without acquiring suppressive function, and FOXP3 intensity/demethylation is rarely annotated in clinical cohorts [6]. Helios (IKZF2) is likewise debated: it correlates with stability in some human Tregs but is not lineage-defining and can be induced outside of thymic Tregs; over-reliance on Helios to call “bona fide” Tregs risks misclassification [73]. These caveats matter when we ask whether specific therapies “deplete Tregs,” or when we derive spatial biomarkers (e.g., ICOS^+^/CCR8^+^ clusters) that guide trials; using composite phenotypes (e.g., FOXP3 with ICOS/CCR8 and functional surrogates) in human HCC samples is preferable [18,45,74]. 

### 8.1. In Vivo Depletion Assessment

Carrying that measurement rigor into the clinic raises a practical question: how much Treg depletion does CTLA-4 blockade achieve in people? Preclinical and translational studies show that anti-CTLA-4 activity can depend on Fc-mediated depletion of intratumoral Tregs; Fc-optimized backbones enhance efficacy via ADCC/ADCP [54].

In the clinic, however, definitive histologic proof of sustained intratumoral Treg loss across human cancers is inconsistent, and for HCC specifically remains limited. Notably, the STRIDE regimen (single high-dose tremelimumab plus durvalumab) improved OS versus sorafenib with acceptable safety without demonstrating biopsy-verified Treg ablation as a mechanism in HCC [17,75]. Whether human CTLA-4 blockade predominantly reconditions Tconv/Teff priming (via dendritic cells) versus physically depleting Tregs is an unresolved question with design implications for next-gen anti-CTLA-4 (Fc-engineered vs signaling-biased) in cirrhotic livers [54].

### 8.2. Methodological Limitations

Methodologically, even our best maps of “where the Tregs are” can mislead. Sampling bias (small cores miss perivascular or capsule-adjacent niches), dissociation skew (fragile eTregs with high ICOS/CCR8 may be under-recovered), and computational artifacts (doublets) can inflate apparent Treg abundance or create chimeric “Treg-like” clusters [76,77,78]. Spatial transcriptomics adds sensitivity but trades resolution and can bleed signals across neighboring spots, complicating CTL:Treg ratio estimates in fibrotic septa and portal tracts [76]. Cohorts also vary in etiology and prior locoregional therapy, each reshaping stromal/myeloid cues that sustain Tregs (e.g., CCL22-CCR4, CCR8, adenosine), so naïve cross-study comparisons are methodologically flawed [12,74,79]. These constraints deserve fuller unpacking because many of the spatial–biological assertions in the Treg literature, including several we have summarized above, rest on platforms whose limits are still being defined. Current spatial-transcriptomic technologies trade off resolution against transcriptome breadth: spot-based platforms (e.g., 10× Visium) capture near-whole transcriptomes but at 55-μm spots that typically contain 3–30 cells, so apparent Treg–CD8 colocalization at the spot level cannot be equated with cell-to-cell proximity and must be deconvolved computationally, with attendant uncertainty in cirrhotic septa where cellularity is high and heterogeneous. Subcellular-resolution imaging platforms (MERFISH, Xenium, CosMx) resolve individual cells but are restricted to pre-selected gene panels of typically a few hundred transcripts, which can miss rare but mechanistically central markers (CCR8, ICOS, NR4A family) unless these are explicitly included and adequately detected. Multiplex protein platforms (CODEX/PhenoCycler, IMC, MIBI) preserve morphology but are capped at tens of antibodies and therefore force a priori choices about which Treg markers to resolve. Second, the human HCC studies we cite differ substantially in tissue preservation (fresh-frozen versus FFPE), region selection (core biopsies versus whole sections), dissociation protocols, and computational pipelines (doublet-removal, batch correction, integration method, annotation reference), each of which can shift the relative abundance and the inferred co-localization of eTreg subsets; some apparent etiology-specific differences may therefore reflect study-design variance rather than biology. Third, derived spatial metrics such as PD-1^+^CD8:ICOS^+^Treg ratios, nearest-neighbor distances, or niche-enrichment scores are highly sensitive to segmentation, field-of-view size, and the inclusion or exclusion of peritumoral/capsular zones; cross-study comparisons without harmonized pipelines remain exploratory. Fourth, spatial distance alone is a weak biomarker: two cells may be adjacent without functional interaction, while distant cells may be coupled by soluble factors or exosomal traffic; inferred cell–cell communication from spatial data should therefore be validated with orthogonal functional assays before it is treated as mechanism. We have tried to flag these uncertainties throughout the review, but we stress that the field would benefit from community-agreed benchmarks, shared reference datasets covering each etiology, and pre-registered analysis pipelines before spatially derived ratios are elevated to companion-diagnostic status. 

### 8.3. Safety Considerations in Cirrhosis

Cirrhosis is an immune-perturbed state with autoimmunity-prone biology; aggressive Treg targeting could amplify immune-related adverse events (irAEs) or hepatic flares. In frontline HCC, atezolizumab + bevacizumab established a survival benchmark with a manageable AE profile, setting the bar for any Treg-directed add-ons [16]. STRIDE improved OS with higher grade ≥ 3 irAEs than durvalumab alone, but maintained overall benefit and patient-reported outcomes [17,75,80]. Future Treg-depleting strategies in Child–Pugh A-B populations must prove they do not convert manageable dermatitis/thyroiditis into decompensation. The hepatic safety calculus for agents that target CCR4 or CCR8 in an already cirrhotic liver deserves specific consideration. Two distinct mechanisms of liver-directed toxicity are plausible. First, HBV reactivation: CCR4 is expressed on a substantial fraction of circulating and tissue Tregs, and clinical experience with the anti-CCR4 antibody mogamulizumab in adult T-cell leukemia-lymphoma has documented HBV reactivation, including at least one fatal case of reactivation hepatitis in a patient with resolved prior HBV infection, despite entecavir prophylaxis [81]. Because HBV-related HCC is by definition a population with either active or resolved HBV, any CCR4-depleting strategy in HBV-HCC must anticipate a non-trivial reactivation risk and incorporate mandatory HBV-DNA monitoring plus nucleos(t)ide analog prophylaxis in all HBsAg-positive patients and anti-HBc-positive patients with detectable DNA. Second, compartment spillover: while CCR8 is substantially more tumor-restricted than CCR4, it is still expressed on a small fraction of thymic Tregs, on subsets of Th2/Th17 cells, and on some tissue-resident memory populations [66]. In a non-cirrhotic liver this may be irrelevant, but in a cirrhotic, autoimmunity-prone liver with pre-existing lymphocytic infiltrates at portal tracts, even modest off-tumor Treg attrition could unmask subclinical autoimmune hepatitis or amplify pre-existing PBC/AIH overlap features. Fc-optimized, ADCC-biased CCR8 depleters are designed to maximize intratumoral activity while sparing peripheral and thymic CCR8^+^ compartments [63,65,82], but human safety data in cirrhotic populations are not yet available. Practical implications for HCC trial design include: (i) stratifying by Child–Pugh class and by viral status at enrolment, (ii) excluding or closely monitoring patients with baseline ALT greater than 3×ULN or active autoimmune liver disease, (iii) mandating HBV and HCV serology plus HBV-DNA at baseline and through treatment, (iv) pre-specifying a hepatic-flare-adjudication process that distinguishes Treg-depletion-related hepatitis from cirrhotic decompensation, ICI-related hepatitis, and viral reactivation, and (v) integrating spatially resolved CCR8/CCR4 mapping of both tumor and background liver to quantify the expected “off-target” compartment that a depleting antibody will encounter.

Finally, etiology shapes both biology and trial design, often decisively. Non-viral, MASH-related HCC harbors dysfunctional, scar-entrenched T cells and may respond less to PD-(L)1 monotherapy; this argues for etiology-aware stratification and for composite metrics that account for suppressive niches rather than bulk TIL counts [53]. Single-cell atlases in HCC already reveal distinct ICOS^+^/CCR8^+^ eTreg aggregates with proximity to exhausted CD8^+^ T cells, a configuration likely to differ by HBV vs MASH backgrounds [12,45,74].

### 8.4. Future Trial Designs

Three translational priorities have emerged from this review.

- CCR8/CCR4-directed depletion with spatial biomarkers. CCR8 marks tumor-resident, highly suppressive eTregs across cancers and is enriched in HCC eTreg clusters; antibodies that exploit CCR8 for selective intratumoral Treg depletion show compelling preclinical rationale and translational feasibility [66]. In models, CCR8 targeting synergizes with PD-1 blockade without systemic autoimmunity; clinical biomarker development should pair CCR8 protein/RNA with spatial “danger zones” (ICOS^+^ Treg-PD-1^+^CD8 adjacency) [45,63,74]. CCR4 recruitment in liver tumors, dual-axis designs (CCR8±CCR4) deserve testing, but require rigorous histologic and single-cell endpoints to confirm selective Treg removal over bystander Th2 [79].
- Adenosine-axis layered onto VEGF + PD-(L)1. Hypoxia, VEGF signaling, and CD39/CD73 converge to generate adenosine that stabilizes Tregs and blunts CD8^+^/NK function. Building on atezolizumab+bevacizumab (and D+B+TACE in intermediate HCC), adding CD73 or A2A blockade could unlock adenosine-mediated non-immunogenic lesions [16,83,84]. First-in-human CD73 inhibition (oleclumab) showed acceptable safety and on-pathway activity with durvalumab, supporting HCC combinations that include explicit adenosine readouts (adenosine metabolites, CD73 occupancy, spatial CD39/CD73 maps) [85]. Trials should prespecify Treg-centric endpoints (CD39^+^/CD73^+^ Treg prevalence; A2A-responsive signatures) alongside classical ORR/OS.
- Etiology-aware trials with composite spatial metrics. Randomization stratified by viral vs metabolic liver disease, with co-primary biomarker endpoints such as PD-1^+^CD8:ICOS^+^Treg spatial ratio and CCR8^+^ eTreg density in tumor centers vs edges, will test whether suppressive topology predicts benefit across regimens (PD-(L)1 alone, STRIDE-like, or VEGF+PD-(L)1±adenosine inhibitor) [45,53,74]. Harmonized pipelines that control for doublets and signal diffusion (and report effect sizes with uncertainty) should be mandated in protocols [76,77,78]

## 9. Materials and Methods

We conducted a targeted narrative synthesis emphasizing human HCC evidence (primary tissues, blood, or clinical cohorts), single-cell/spatial studies, and interventional trials through September 2025. Sources were identified via PubMed and Embase using combinations of “hepatocellular carcinoma,” “regulatory T cell,” “FOXP3,” “CCR8,” “ICOS,” “CD39,” “CD73,” “single-cell,” “spatial transcriptomics,” and “trial/atezolizumab/bevacizumab/durvalumab/tremelimumab.” Inclusion prioritized peer-reviewed journals. Animal data are incorporated solely to contextualize human findings where necessary. 

## 10. Conclusions

The intrinsic tolerance of the liver and the immunosuppressive microenvironment of HCC necessitate therapeutic strategies that go beyond simple checkpoint blockade. While current standard-of-care regimens, such as atezolizumab plus bevacizumab and tremelimumab plus durvalumab, achieve clinical benefit, their impact on regulatory T cells is largely indirect or compartment-specific rather than selectively intratumoral. Consequently, significant opportunity remains to deepen responses by specifically targeting the effector Treg niches that persist despite PD-(L)1 blockade.

The integration of high-resolution single-cell and spatial profiling has clarified that not all Tregs are equal targets; specifically, the CCR8^+^ effector Treg subset represents the most promising biomarker for identifying suppressive tumor niches. Correspondingly, CCR8-directed depletion and adenosine-axis blockade (targeting CD73/A2A) emerge as the most viable next-generation therapeutic strategies to selectively dismantle these hubs without inducing systemic autoimmunity. Future clinical success will depend on validating spatially derived ratios (such as PD-1^+^CD8:ICOS^+^Treg) as companion diagnostics and stratifying patients by etiology to maximize the precision of these combinations.

Take-home messages. Five points crystallize from this review. (1) The clinically relevant Treg population in HCC is not the bulk FOXP3^+^ compartment but the intratumoral effector Treg (eTreg) subset defined by co-expression of CCR8, ICOS, CTLA-4, and the CD39/CD73 ectonucleotidase pair; human studies, not murine surrogates, should drive target selection, and where possible TSDR methylation or composite CD45RA/FOXP3/CCR8/CD39 phenotyping should replace FOXP3 alone. (2) Spatial context is prognostic and predictive: low intratumoral CD8/Treg or PD-1^+^CD8:ICOS^+^Treg ratios identify suppressive niches that track with worse outcomes and with reduced ICI benefit; these composite ratios are the logical candidates for companion diagnostics if analytical pipelines can be harmonized. (3) Etiology matters: HBV-related HCC organizes Treg–Tex niches around viral antigens through shared NFAT/NR4A/NF-κB programming, whereas MASH-related HCC operates through lipotoxic, stromal, and metabolic barriers that can blunt PD-(L)1 efficacy; trial designs that ignore this distinction will continue to produce heterogeneous results. (4) Standard-of-care backbones (PD-(L)1 ± anti-VEGF; STRIDE) already touch Treg biology indirectly, but the near-term opportunity lies in layering selective, intratumor-biased Treg modulation—CCR8-directed depletion, CCR4-directed depletion where justified, ICOS-guided pruning, and adenosine-axis blockade—onto these backbones, with explicit on-target tissue readouts. (5) Cirrhosis constrains the therapeutic window: HBV reactivation (particularly with CCR4 depletion) and off-tumor Treg spillover in an autoimmunity-prone liver must be pre-empted by viral screening, nucleos(t)ide prophylaxis, Child–Pugh stratification, and hepatic-flare adjudication processes built into trial design. Collectively, these points argue for an etiology-aware, spatially biomarker-guided, liver-safety-conscious framework as the most rational path from current Treg biology to clinically meaningful HCC outcomes.

## Figures and Tables

**Table 1:** Mechanisms of Treg recruitment and maintenance in HCC [12,34,35,36,37].

Mechanism	Key Molecules	Function	Therapeutic Implication
Recruitment	CCL20-CCR6	Chemotaxis from blood/peritumor to tumor.	Blockade may reduce initial Treg influx.
	CCL22/17-CCR4	Recruitment into macrophage/DC-rich niches.	CCR4-depleting antibodies (e.g., Mogamulizumab).
	CCL1-CCR8	Positioning of highly suppressive eTregs.	CCR8-specific depletion (e.g., BAY3375968).
Maintenance	TGF-β	Differentiation and stability of FOXP3 expression.	TGF-β traps; bifunctional antibodies.
	IL-10	Maintenance of suppressive phenotype via STAT5.	JAK/STAT inhibitors; IL-10 blockade.
	Adenosine (CD39/CD73)	Metabolic suppression; stability in hypoxic TME.	A2A receptor antagonists; anti-CD73.
Suppression	CTLA-4	Trans-endocytosis of CD80/86; inhibitory signaling.	Anti-CTLA-4 (e.g., Tremelimumab).
	IL-35	Propagation of suppressive capacity (infectious tolerance).	IL-35 neutralization (experimental).
	IL-2 Sequestration	CD25-high Tregs deprive effectors of IL-2.	IL-2 muteins (biased to CD8/NK cells).

**Table 2:** Impact of disease etiology on Treg recruitment, metabolic niches, and immunotherapy response in HCC [11,12,18,34,35,36,37,38,39,40,41,42,43,44,45,46,47,49].

Etiology	Dominant Context	Treg Recruitment Axes	Metabolic Niche	ICI Response Pattern (Qualitative)	Trial Design Implication
HBV-HCC	Viral antigens; chronic inflammation	CCR4/CCR8; VEGF-linked	Hypoxic cores → adenosine	Often responsive with selection	Capture HBV therapy status; include adenosine-axis
HCV-HCC	Post-DAA legacy	Similar to HBV, attenuated after cure	Variable	Intermediate	Stratify by cure timing
MASH-HCC	Lipotoxicity; fibrosis	CCR2/CCR5 myeloid crosstalk; CCR4/CCR8 variable	Lactate-rich; FAO-skewed Tregs	Blunted in subsets	Pair ICIs with metabolic/adenosine targeting
Alcohol-related	Oxidative stress; gut-liver axis	Heterogeneous gradients	Hypoxia/necrosis	Variable	Control for ongoing injury

HBV: hepatitis B virus; HCV: hepatitis C virus; MASH: metabolic dysfunction-associated steatohepatitis (within MASLD); MASLD: metabolic dysfunction-associated steatotic liver disease. FAO: fatty-acid oxidation; ICI: immune checkpoint inhibitor; DAA: direct-acting antiviral. CCR/CCL: CC-chemokine receptor/ligand families (e.g., CCR2/CCR5, CCR4/CCR8). Symbol “→” denotes “promotes” or “drives toward.

**Table 3:** Therapeutic targets modulating the Treg axis: mechanisms, representative agents, and clinical implications [11,13].

Setting/Backbone	Target	Mechanism	Representative Agent	Clinical Implication
**1L advanced** (PD-(L)1 + anti-VEGF)	PD-(L)1 + VEGF axis	VEGF blockade reduces Treg trafficking/retention; PD-(L)1 reinvigorates CD8^+^	Atezolizumab + Bevacizumab	↑ CD8/Treg; vascular normalization enhances immune infiltration
**1L advanced** (PD-(L)1 + CTLA-4)	CTLA-4	FcγR-dependent intratumoral Treg depletion or reprogramming	Durvalumab + Tremelimumab (STRIDE), Nivolumab + Ipilimumab	↑ CD8/Treg (compartment-specific); variable Treg depletion
**1L/2L** (TKI monotherapy)	Anti-angiogenic TKIs	Indirect cytokine/angiogenic modulation of Treg trafficking	Lenvatinib, Sorafenib	↔ /mild ↑ CD8/Treg; limited direct Treg effects
**2L+** (PD-(L)1 monotherapy)	PD-(L)1	Effector reinvigoration without direct Treg depletion	Pembrolizumab, Nivolumab	↔ CD8/Treg; context-dependent
**Any**	CCR8	Selective depletion of intratumoral eTregs; spares peripheral Tregs	Anti-CCR8 mAbs (humanized IgG1)	↑ CD8/Treg; high specificity for tumor-resident Tregs; strong synergy with PD-(L)1
**Any**	CCR4	Blockade of CCL17/CCL22-mediated Treg recruitment	Mogamulizumab	Reduces CCR4^+^ Treg influx; may synergize with PD-1 inhibitors; risk of peripheral Treg loss
**Any**	ICOS	Targeting ICOS^+^ eTregs in suppressive niches	Anti-ICOS antibodies (agonistic or depleting)	Modulates ICOS^+^ Treg clusters; biomarker-guided potential; dual Treg/effector effects
**Any**	Adenosine axis (CD39/CD73 → A2A)	Inhibition of adenosine-mediated metabolic suppression of CD8^+^ T cells	CD73 inhibitors, A2A antagonists, dual CD39/CD73 blockers	Restores CD8^+^ function in hypoxic TME; ↑ functional CD8/Treg; strong rationale for combinations

PD-(L)1: programmed cell death protein-1/programmed death-ligand 1 inhibitor; VEGF: vascular endothelial growth factor; CTLA-4: cytotoxic T lymphocyte-associated protein-4; STRIDE: Single Tremelimumab Regular Interval Durvalumab; nivo/ipi: nivolumab/ipilimumab; TKI: tyrosine-kinase inhibitor; adenosine axis: A2A/CD73/CD39 pathway; CCR8/CCR4: chemokine receptors enriched on tumor-resident Tregs; ICOS: inducible T-cell co-stimulator, enriched on eTregs in suppressive niches. Definition: “Expected net effect on CD8/Treg” = qualitative directional change in intratumoral cytotoxic CD8^+^ T cells relative to Tregs. Symbols: ↑ increase; ↔ no meaningful change; mild ↑ small increase; “compartment-specific” = effect differs between tumor, peritumoral, or lymphoid sites.

## Data Availability

No new data were created or analyzed in this study. Data sharing is not applicable to this article.

## Abbreviations

4-1BB	CD137 is a co-stimulatory receptor on T cells.
A2A	Adenosine A2A receptor.
AE	Adverse event.
APC	Antigen-presenting cell.
CCL	CC-chemokine ligand.
CCR	CC-chemokine receptor.
CD25	IL-2 receptor α chain.
CD36	Long-chain fatty-acid transporter.
CD39 (ENTPD1)	Ectonucleotidase generating adenosine.
CD73 (NT5E)	Ecto-5′-nucleotidase generating adenosine.
CTLA-4	Cytotoxic T lymphocyte-associated protein-4.
DAA	Direct-acting antiviral.
DC	Dendritic cell.
eTreg	Effector regulatory T cell.
FAO	Fatty-acid oxidation.
FcγR	Fc-gamma receptor.
FOXP3	Forkhead box P3.
GARP	Glycoprotein A repetitions predominant.
HBV	Hepatitis B virus.
HCC	Hepatocellular carcinoma.
HCV	Hepatitis C virus.
HSC	Hepatic stellate cell.
ICI	Immune checkpoint inhibitor.
ICOS	Inducible T-cell co-stimulator.
IL-10	Interleukin-10.
IL-35	Interleukin-35.
irAE	Immune-related adverse event.
JAK1/STAT5	Janus kinase-1 / Signal transducer and activator of transcription-5.
LSEC	Liver sinusoidal endothelial cell.
MASLD	Metabolic dysfunction-associated steatotic liver disease.
MASH	Metabolic dysfunction-associated steatohepatitis.
MCT1	Monocarboxylate transporter-1.
NFAT	Nuclear factor of activated T cells.
NF-κB	Nuclear factor κB.
NK	Natural killer (cell).
NRP1	Neuropilin-1.
OX40	TNFRSF4; co-stimulatory receptor.
OXPHOS	Oxidative phosphorylation.
ORR	Objective response rate.
OS	Overall survival.
PD-1	Programmed cell death protein-1.
PD-L1	Programmed death-ligand-1.
PD-(L)1	PD-1 or PD-L1 inhibitor (umbrella term).
PFS	Progression-free survival.
PPAR-β	Peroxisome proliferator-activated receptor-β.
RA	Retinoic acid.
rTreg	Resting regulatory T cell.
scRNA-seq	Single-cell RNA sequencing.
STRIDE	Single Tremelimumab Regular Interval Durvalumab regimen.
TACE	Transarterial chemoembolization.
Tex	Exhausted T cell.
TIL	Tumor-infiltrating lymphocyte.
TKI	Tyrosine-kinase inhibitor.
TME	Tumor microenvironment.
VEGF	Vascular endothelial growth factor.
